# HIV/AIDS clients, privacy and confidentiality; the case of two health centres in the Ashanti Region of Ghana

**DOI:** 10.1186/s12910-016-0123-3

**Published:** 2016-07-16

**Authors:** Jonathan Mensah Dapaah, Kodjo A. Senah

**Affiliations:** Department of Sociology and Social Work, Kwame Nkrumah University of Science and Technology, Kumasi, Ghana; Department of Sociology, University of Ghana, Legon, Accra Ghana

**Keywords:** HIV/AIDS, Social risks, Confidentiality, Privacy, Counselling, Treatment, Stigma, Sero-positives

## Abstract

**Background:**

While most studies on HIV/AIDS often identify stigmatization and patients’ unwillingness to access health care as critical problems in the control of the pandemic, very few studies have focused on the possible consequences of accessing health care by sero-positives. This paper examines the socio-psychological trauma patients experience in their desire to access health care in two health facilities in the Ashanti Region of Ghana.

**Methods:**

Through participant observation, informal conversation and in-depth interviews, data were collected from health workers and clients of the voluntary counselling (VCT) and antiretroviral therapy units in the two hospitals.

The data gathered were analysed and categorized into themes and supported with illustrative quotes obtained from health workers and clients.

**Results:**

The study found that the mere presence of a person at the HIV counselling centre or clinic is enough for the person to be labelled as or suspected to be HIV patient. It demonstrates that stigmatization may occur not only in the community but also overtly or covertly, in the health facility itself. Consequently, for many HIV/AIDS patients, access to antiretroviral therapy and treatment of related nosocomial infections are problematic. Besides, the study found that many clients and potential users of services were uncomfortable with the quality of care given by some health workers, especially as they overtly and covertly breached confidentiality about their clients’ health status. This has compelled many patients and potential users of the services to adopt a *modus vivendi* that provides them access to some care services while protecting their identity.

**Conclusion:**

The paper argues that by examining issues relating to privacy and confidentiality in the provision of care for and use of services by seropositives, more light will be shed on the whys of the limited uptake of HIV-related health care services in Ghana.

**Electronic supplementary material:**

The online version of this article (doi:10.1186/s12910-016-0123-3) contains supplementary material, which is available to authorized users.

## Background

*Yes, it is true that some people have cited lack of confidentiality on the part of health workers as a reason for not using HIV-related health care services. Some of our colleagues talk too much … so they easily disclose the status of clients forgetting that this may lead to stigmatization of the clients. Other clients have also complained about the open location of the clinic in this hospital… They fear that relatives may see them entering or coming out of the clinic and suspect them of the disease, and stigmatise them* (Marie, a health assistant).

The introductory quotation (above) provides the kernel for this paper: sero-positives desirous to access health facilities have two psychological battles to fight—first, against friends and relatives who must not know of his/her HIV status and second, against healthcare providers entrusted with confidential information on their health status. In making the decision to access HIV counselling, testing and treatment services, clients are often concerned about the possibility of a breach of their status and the negative repercussions or risks that may follow. Lyimo et al. [1] pointed out that the risks relate to people’s beliefs and perceptions about the disease because in spite of several years of public education, HIV/AIDS is still considered contagious, severe, life-threatening and presumably the result of a norm-violating behaviour such as commercial sex work, homosexuality and promiscuity. In Ghana, these risks are often culturally constructed as shameful (*animguaseè*), loss of respect (*onnibuo*) and dishonourable (*onnianimuonyam*). Kwansa [2] and Dapaah [3] observed that the consequences of these include divorce, rejection, ostracization, discrimination and loss of job. According to Mbonu et al. [4], in sub-Saharan African countries, including Ghana, the fear of stigma and its repercussions negatively affect potential clients seeking HIV testing, diagnosis and treatment. This situation is of grave concern to stakeholders in the campaign to prevent the spread of HIV/AIDS.

To reduce stigma and encourage more people to go for counselling, testing and treatment, a scale-up exercise was carried out in Ghana between 2005 and 2010. This exercise led to phenomenal increase in treatment sites and as at the end of 2013, there were 324 VCT centres and 26 ART clinics in the Ashanti Region where this study was carried out. Campbell et al. [5] noted that the scale-up exercise was largely influenced by the argument that as ART becomes universally accessible, stigma will gradually disappear.

However, interactions with some health workers pointed to the contrary: they observed that the increase in the number of treatment sites has not led to the expected rise in the use of services and the subsequent reduction in the prevalence of HIV/AIDS stigma. This observation finds parallel in a study by Campbell et al. [5] which pointed out that to date, the restoration of users’ own sense of self-worth through treatment as not reduced fear and sexual embarrassment in framing communities’ responses to people living with HIV/AIDS in Zimbabwe. As can be appreciated from the opening quotation above, Marie, a health assistant, attributed the low uptake of the services by clients to two significant factors: first, fear of breach of confidentiality by some health workers leading to the stigmatization of clients; and second, the open location of the clinics, a situation that does not guarantee clients the privacy to access services and conceal their status from others. These concerns relate to confidentiality and privacy in the health care setting.

The theory underpinning this article is agency. The persistent stigma associated with HIV infection makes it imperative for clients to devise strategies and secretly use counseling, testing and treatment services. This will enable them to conceal their status from relatives to friends and also prevent stigmatization. To understand how clients manage the fear of stigma related to the up-take of VCT and ART services, clients are contextualized as actors who have the capacity to act and take decisions to use services in the facilities in safety. Thus, the article is interested in how clients appraise the risk of being exposed as positive persons against the benefits they will derive from accessing services, leading to a particular choice of action or inaction. The term agency, then, according to Giddens [6] suggests intention or consciousness of action, sometimes with the implication of possible choices between different actions. Patients do possess the ability to act and make choices on the use of services. Therefore, the article illustrates how clients act and strategize to access services in various locations in the hospitals and avoid identification as would be seen in the ethnographic data and discussion presented below.

## Methods

### Study population

The study on which this article is based was part of a larger project made up of three interrelated studies on the provision and use of VCT and ART in Ghana. The first study focused on the social and cultural factors that discourage or inhibit HIV-positive persons and other potential clients from using VCT and ART services. It was carried out in two health facilities providing voluntary counselling and testing (VCT) and antiretroviral (ARV) therapy. The hospitals were purposively selected for the study because they were among the few health facilities in the Ashanti Region which benefited from the initial scale-up of VCT and ART in Ghana. The second study was conducted in the communities served by the two hospitals; its focus was on the social and cultural constructions of blame, shame and stigma. The third project examined the policy-making processes on international, national and local levels and how these facilitated or constrained the use of VCT and ART services in Ghana.

For this study, health workers were the primary target group and over 40 of them were involved in the study. Some of the care providers were purposively selected for interview based on a set criteria. The criteria included: (i) the position of the health worker in the counselling centre or HIV clinic; (ii) at least 2 years working experience in the facility; and (iii) willingness to participate in the study. The researchers were of the view that a health worker who satisfied these basic requirements could effectively answer questions on the core issues of the study. Over 100 clients participated in the study as the secondary target group. Similarly, selection of some clients for interview was also based on the following criteria: (i) health condition; and (ii) willingness to be interviewed. This requirement for clients was motivated by the fact that those who looked very sick and traumatised by HIV-positive diagnosis would be unwilling to talk about the challenges they were going through.

### Methods of data collection

Data were compiled using qualitative research methods including participant observation in the diverse counselling, testing and clinical settings where clients accessed care and treatment, informal conversations and in-depth interviews. These approaches were complemented with a review of hospital records and socio-demographic characteristics of clients. During field research, the lead author participated in daily activities in the clinic and observed interactions between health workers, clients and peer educators. For instance, he participated in testing and counselling sessions and consultations and observed interactions between counsellors and clients. In the processes the researcher engaged in conversations with health workers, clients and their relatives to get their views on provision and use of services. Unlike formal interviews, conversations with health workers and clients were more informal and spontaneous and were therefore less likely to impel a participant to say what would please the researcher. Finally, with the use of unstructured interview guide, the first author was able to acquire insightful accounts from health workers and clients on the provision and use of the services.

More than forty (40) health workers and over 100 clients in the VCT centres and ART clinics were observed. In all, 24 health workers who provided counselling, testing and treatment were selected and interviewed, including six nurses, five medical doctors, one pharmacist, one pharmacist technologist, two laboratory technicians, one disease control officer, five counsellors, one health assistant and two cleaners. Twenty-two clients were also interviewed, made up of twelve women and ten men. The data collection instrument was an interview guide as described below under Additional file 1 after references.

### Data management and analysis

Interviews were recorded and transcribed by the researcher. The transcribed interviews were carefully read through and broken down into meaning units and coded. The codes were grouped in broad categories that were defined and constructed on the basis of the data collected. A summary was made for each category building on the common and recurrent responses as well as on different view points. The categories with the summaries and the coded meaning units were identified and illustrative quotations were selected. The two broad themes which were identified from the data as influencing clients use’ of HIV-related health care services included positive and negative attitudes and behaviours of health workers.

## Results

### Confidentiality

Counselling is the first step in the uptake of HIV/AIDS-related health care services. According to IEA/UNFPA [7], counselling is “a dialogue between a client and a counsellor which aims at enabling the client to cope with stress and take personal decisions related to HIV/AIDS”. To start a normal counselling session, counsellors always assured clients of confidentiality; they assured clients that other people would not know about any discussion or conversation between the two of them. This implies that the information gathered from patients must be managed in such a way as to keep it from other people. Confidentiality thus establishes trust between the two parties because it enables clients to conceal their status from relatives and others and avoid the social costs associated with the disease.

In some cases, however, clients allowed health workers to disclose their status to selected persons in the context of shared confidentiality or couple counselling. Yet, some clients accused some health workers of a breach of the concordat during counselling. As the clients pointed out, in as much as they believe in the efficacy of the ARVs, they were rather afraid that other people might know about their status through the health workers. Interestingly, some health workers, agreed with this assertion by clients. Some health workers, however, distinguished between two types of breach of confidentiality: unintentional and intentional.

#### Openly talking about clients’ status (unintentional disclosure)

These were cases when some health workers were said to have revealed the status of clients to their own colleagues in other units of the hospitals in line with hospital administrative procedures. Both health workers and clients cited instances to support this point. Rebecca, a senior nurse recalled how a colleague in conversation with another health worker at the waiting section of the hospital openly disclosed the status of a client to the hearing of other patients in the waiting section. The nurse said that in order to protect the identity of sero-positive, health workers call them ‘clients.’

Awo, a client, also told of an instance in which a nurse openly disclosed her status and that of another colleague to another health worker as they (the clients) were being shown the way to the ART clinic. According to Awo, the moment the nurse unguardedly disclosed their status in the corridor of the hospital, other patients waiting for treatment suddenly turned and gazed at them. Similarly, Salifu, a client, explained that after the doctor had finished attending to him, the doctor called a ward assistant and openly asked her to take him (Salifu) to where HIV patients are treated. According to Salifu, the doctor said this to the hearing of other patients waiting for treatment at the outpatients department. He added further:

*The moment I came out of the doctor’s room with the ward assistant, everybody was looking at me. I observed that some of them were gossiping about me. I did not hear what they said but I believe they were saying that I am HIV-positive based on what they heard the doctor said.*

Throughout the fieldwork, the authors were confronted with several instances which questioned critical aspects of medical/health ethics. Health practitioners were highly paternalistic often to the point where patients’ responsibility and autonomy were literally whittled away from their hands. This is not new; in the Ghanaian clinical encounter, the frequency of depersonalization of the patient with its attendant abuse of patient rights led to the introduction of the Patient’s Charter by the Ghana Health Service in the mid—2000. This charter is a compendium of the rights and responsibilities of patients and the general moral principles and rules of behaviour for all public health personnel. In more specific terms, section (b) of the code of ethics for health personnel stipulates: All service personnel shall respect the right of patients/clients, colleagues and shall safeguard patients/clients’ confidence. In affirmation of this, section (f) of the code states: All service personnel shall respect confidential information obtained in the course of their duties. They shall not disclose such information without the consent of patient or person(s) entitled to act on their behalf, except where the disclosure is required by law or is necessary in the public interest. It is therefore worth noting that such disclosures as observed in the present study can erode the trust clients have in health workers and keep clients away from the health centres.

#### To disclose or not to disclose? (intentional disclosure)

It was found that health workers who handle HIV/AIDS clients often find themselves in a very difficult situation: clients are not handled solely by health workers but also by significant others who require information on clients’ status to be able to assist with care. This situation clearly presents a jigsaw puzzle for health workers who often opt to inform the latter with or without the consent of the client. These significant others included spouses and some close relatives. Clients told of their experiences of how health workers informed their spouses and relatives about their status without first seeking their approval and the consequences thereof.

Amma, a client, initially denied her status when the husband observed that she was taking medicines morning and evening on a daily basis. She lied that the medicines were for the control of high blood pressure. However, not convinced, her husband stealthily took Amma’s medicines and treatment cards to the clinic and showed them to a nurse who explained that the medicines and treatment cards belonged to a sero-positive. This disclosure not only led to the break-up of Amma’s marriage but also Amma’s husband went further to inform her family members that she was HIV-positive. Consequently, Amma blamed her predicament on the health worker who disclosed her status without her consent. She opined in a conversation:

“*I did not inform him (my husband) of my status because I feared that could lead to divorce but this has eventually happened through a health worker.”*

Furthermore, during this study, a health worker disclosed a client’s status to some community members and the client threatened to stop accessing services. The client, Bruwaa, explained that a health worker had gone public about her status by warning her lover to stay away from her because she is HIV-positive. According to her, since then community members have been gossiping about her status.

Nurse Suzane, however, explained that health workers sometimes need to disclose the status of clients to others in the interest of the clients themselves or their spouses. For, instance, she explained that in some cases, health workers cannot just look on and allow infected mothers to infect their babies with HIV through breast feeding. Suzane further explained that the only way to prevent this is by involving spouses or relatives in the counselling process to enable them understand why a lactating mother has been asked not to breastfeed her baby. Doing this necessarily leads to intentional status disclosure.

However, there were instances in which health workers often helped clients to disclose their status to spouses. In a few cases, ‘such assisted’ disclosures have led to problems between spouses resulting in marriage break-up.

### Privacy and the use of services

According to HRSA [8], privacy refers to an individual’s right to control both access to and use of his or her health information. Similarly, Burn and Grove [9] posits that privacy is the freedom an individual has to determine the time, extent and general circumstances under which private information on him/her will be shared with or withheld from others. For the purposes of this paper, privacy refers to the practice of keeping clients’ personal data or information away from others as well as ensuring that the reason for a client’s physical presence in the health facility is not known by other people. It is in this regard that Marie’s comment (at the beginning of this article) shows the importance clients attach to accessing care and treatment in privacy. Health workers observed that due to persistent stigma associated with the disease, clients preferred to access services in privacy in order to avoid being identified by relatives or acquaintances. Clients also said that using services in privacy enables them to conceal their status from other people. In this respect, health workers and clients identified some structural or institutional challenges in the health care setting which do not guarantee privacy in the use of services. Some of these are identified below.

#### Location of centres and clinics

Most clients expressed concern about the open and stand-alone locations of some facilities which do not guarantee privacy while accessing care and treatment. They often feared that other people might see them entering or exiting the facilities and suspect them of HIV infection. According to them, the moment they get to the hospital premises, their attention is shifted from care and treatment to employing strategies to prevent relatives and acquaintances from seeing them near or inside the treatment facility. In one of the study hospitals for instance, the open location of the HIV/AIDS facility immediately exposed all persons-patients and visitors-to the glare of everybody. This is because the HIV clinic is closely located at the main entrance to the hospital.

Clients therefore employed ways and means to access services in such openly located facilities *in cognito*. One client remarked as follows:

*”Whenever I am entering the clinic or coming out, I always look around and make sure that somebody I know does not see me.”*

In the other facility, the location of the counselling and testing centre is easily accessible to mothers attending postnatal care (PNC), administrative staff and visitors to some offices of the hospital. Clients said that the location of this centre make them feel insecure while accessing counselling and testing because mothers or visitors can see them and suspect them of the disease.

#### Modes of using services

Clients expressed concerns about certain arrangements for provision of services which isolate them from other patients.

In one of the facilities, pre and post-test counselling sessions were done in one room while testing was done in another. After pre-test counselling, clients joined a queue for testing in another room adjacent to the counselling room. They came back to the counselling room after the test for the disclosure of the results and post-test counselling. This was different from what I observed in most facilities where counselling and testing were done in the same room. Some health workers and clients pointed out that the shuttling between the counselling and testing rooms could undermine clients’ privacy. The movement between the counselling and testing rooms occurred in the full glare of mothers attending postnatal care and visitors to the offices close to the counselling and testing room.

In this same hospital, clients accessed various laboratory services in different units with other patients. The CD4 count test was done in one unit of the hospital near the admission wards while the haemoglobin test was done in another laboratory close to the outpatients department in a different building. Tuberculosis tests were, however, done in a laboratory within the ART clinic. Clients told of how the movement from one unit to another for services could possibly expose their status to other patients.

- The Adherence Monitor:
  - In Ghana, one of the conditions for accessing ART services is for a client to appoint an ‘adherence monitor.’ This is supposedly a trusted person a client informs about his/her status and the monitor in turn helps him/her to adhere to treatment regimen. The adherence monitor is a mandatory inclusion criterion for starting treatment. It is also an aspect of shared confidentiality aimed at helping clients to adhere to ARV medicines. Health workers and clients expressed concerns about the lack of privacy regarding the involvement of adherence monitors in pre-and post-counselling. Health workers explained that although they are aware that adherence monitors can reveal clients’ status to others, they cannot do anything about it. According to Brenya, a client, the difficulty of many clients is the fact that they have to reveal their status to the monitor before presenting him for adherence counselling. This means that the monitor could tell other people about the client’s status before they come to the clinic for counselling. Brenya cited an instance in which a friend’s adherence monitor spread news about his status and this made the client’s life uncomfortable in the community. In the attempt to keep their status hidden from their spouses and family members, some clients presented people they did not have any relationship with as adherence monitors. Such people agreed to accompany clients to the clinic as adherence monitors on humanitarian grounds sometimes without monetary inducements. In most of such cases, clients managed to convince such people to accompany them to the hospital and stand-in as witnesses for their medical treatment. They therefore agreed to the request of the clients and came to the clinic with them only to learn from the nurses that they have been deceived.
- Admission wards:
  - Health workers and clients were not happy with the lack of admission wards for positive persons in the hospitals. In particular, those in the clinics said that putting HIV-positive persons in the same wards as other patients has been the cause for the poor nursing care and treatment they received while on admission. They said that most of the time nurses in the wards gave less nursing care to HIV clients than to other patients. Besides, in one of the hospitals some nurses put these clients at the side wards in order to have less contact with them. This, according to the health workers has often exposed the clients as HIV-positive persons in the wards. Vera, a senior nurse, took one of the researchers round a ward where some clients were on admission to prove this assertion.
  - Vera, the nurse said that staff in the clinic have suggested to the hospital authorities to allocate one particular ward to their clients who go on admission as in the case of Korle-Bu Teaching Hospital in Accra, the premier hospital in Ghana. They also suggested that such a ward should be staffed with health workers who have been trained to provide care and treatment to positive persons like those of them working in the VCT centres and ART clinics. They believe this would help clients to receive good nursing care on admission.
  - Carolina, a senior nurse in the other hospital, agreed with Vera’s concerns about seropositives in general admission wards. She pointed out that in her hospital, most HIV-positive mothers left the maternity ward after they have been discharged with their status known to other mothers. Carolina said that health workers in the maternity ward sometimes openly discussed the status of such mothers to the hearing of other mothers during conversations. However, the nurse said that she does not think allocating a separate ward to HIV-positive persons would help them to conceal their status or avoid all the problems associated with the disease. Carolina added that experience has shown that when the unit for a chronic disease is sited within or close to another unit of the hospital, it helps to prevent the facility from being stigmatised by both health workers and patients.
  - Clients on their part narrated many instances of what happened in the admission wards that exposed their status to other patients. Kantanka, a client, said that after nurses in the ward where he was on admission realized that he was HIV-positive they refused to come closer to his bed located in a far corner, which is often referred to as the ‘side ward.’ Besides, a nurse attended to him wearing double pair of hand gloves although she did not wear gloves while attending to other patients close to his bed. According to the client, after the nurse left his bed, he heard two other patients discussing his possible status as evidenced by the double gloves the nurse wore before attending to him.

## Discussion

From the discussion so far, it is obvious that stigma is the key underlying reason for the reluctance of many people to take up voluntary counselling and testing leading to antiretroviral therapy treatment. According to Niehaus [10], the main cause of stigma is the association of the disease with death rather than sexual promiscuity. Writing about South Africa, he acknowledges that this assertion is not entirely new but it shifts the emphasis for the cause of HIV-related stigma from immoral sexual behaviour or unprotected sex to fear of death. He observes that although immoral sexual behaviour is frowned upon by society, the sentiments against it are not strong enough to lead to the persistent stigma related to the disease and positive persons. In some African cultures, men are allowed to marry more than one woman and teenage pregnancies due to pre-marital sex are also common. According to Burn and Grove [9], this perception of HIV infection illuminates many aspects of people’s responses to the disease, including the (non) use of health care services. It also helps to explain why most clients preferred to use services in privacy and hide their status from relatives and others.

These views about the disease are common in Ghana. In a study on the disease among the Akan in Ghana, Crentsil [11] observes that positive persons are often stigmatised because the disease is believed to be incurable and the fear that the infected person is likely to die of it. Likewise, the pain and suffering the person goes through before death is generally considered as shameful and disgraceful, and in some cases lead to perfunctory funerary rites. This is often due to the fear of contagion associated with the corpse of person who died of HIV infection. Such death is described as ‘bad death.’ The concerns of clients in this study on the risks associated with the disease resonate Niehaus [10] and Crensil’s [11] findings that HIV is a deadly disease. The fear of death associated with the disease and the shame that often characterises HIV-related death could frighten relatives and others to distance themselves from its sufferers. It is therefore not surprising that most clients in this study said that their relatives would reject or ostracise them if they got to know of their status.

Confidentiality is central in the communication between health workers and clients in the provision of counselling, testing and treatment services. The professional ethics of health workers obliges them to keep information obtained in contacts with clients to themselves, as stipulated in the Ghana Draft Policy Document on HIV/AIDS by MOH/UG [12]. This was also the rule in the facilities where the study was carried out. Maintaining confidentiality by health workers in matters relating to HIV infection, care and treatment is therefore about trust. However, the trust clients have in health workers may be eroded where it is promised and not fulfilled. The possible lack of confidentiality in the health care setting, on the other hand, is a constraint on the use of services and discourages clients and potential clients from accessing counselling testing and treatment services.

Although health workers acknowledge that confidentiality is core to the provision of counselling, testing and treatment, they see the possible harm of strictly enforcing confidentiality with unintended risks for others. It places them in a dilemma as to whether they should maintain confidentiality or not when they know that a client is likely to infect others. As Nuwagaba-Biribonwoha et al. [13] point out, the dilemma of nurses regarding patients who feel unable to disclose their test results and who may go home to infect partners and unborn children is that they struggle with their professional ethical obligation to maintain confidentiality. In this study, health workers encountered the same problem. Health workers face the dilemma of either observing the rights of clients to maintain confidentiality as required or ignoring this right and breaching confidentiality. Maintaining confidentiality becomes more complicated when health workers are expected to help prevent the spread of the disease by providing information on behaviour change to the infected and uninfected.

It follows that some health workers in this study necessarily disclosed clients’ status to others such as spouses or relatives without their consent. Health workers did that in the interest of clients, particularly in the case of infected mothers. The spouses or relatives of these mothers were involved in counselling on the need for such mothers not to breastfeed their new-born babies. Such disclosures were motivated by the belief that those relevant others would help clients to adhere to treatment or also present for counselling and testing. However, some of these mothers held the view that the nurses did that to embarrass them for voluntarily disclosing their status to relevant others. Others also blamed the nurses for making such disclosures which negatively led to marital difficulties It is in view of these challenges that clients are often encouraged to go for couple counselling, shared confidential or arranged disclosure through which health workers helped many of them to inform their spouses and relevant others about their status. This, in most cases helped to reduce the negative repercussions associated with positive test results in the case of women and led to the use of treatment services. One study in Kenya by Farquhar [14] similarly showed that pregnant women who were offered couple counselling with partners were three times likely to report using ART for prevention of mother-to-child transmission. Another study by the South India AIDS Action Group (SIAAP) [15] in Tamil Naadu reported that couple counselling schemes have shown success in reducing the levels of violence and the numbers of abandoned women after diagnosis. Obviously, the fact still remains that in most of the cases, health workers disclosed the status of some clients in the interest of public good.

The policy of maintaining confidentiality also has wider implications for a client’s therapy management group and the health worker-patient relationship. In most cases, especially in the Ghanaian tradition, relatives and sometimes neighbours often play a significant role in the care and treatment of a sick person as cultural responsibility. In fact, these individuals sometimes come together as a group and take all decisions regarding care and treatment on behalf of the patient. The group’s responsibility goes beyond the popular and folk medicine and extends to the hospital setting. It is for this reason that Gruskin et al. [16] report that in many sub-Saharan African countries, patients are often accompanied to health care settings by family members. Thus, ward environments and clinics tend to be characterized by over-crowding and lack of space for private discussions (see also Mulemi [17]). In his study on the search for therapy in Zaire, Janzen [18] refers to these people as the ‘therapy management group’ of the patient. He characterises the group as mainly a set of close kin members who help with the management of illness or therapy for a kin member. Members of the group thus know the type of sickness or disease the patient is suffering from in order to take appropriate decisions on cure and treatment, either in the hospital or in the folk system (see also Bossart [19]). It also implies that health workers cannot maintain confidentiality about the patient’s health problem since some relatives, as therapy managers of the patient must always be present during consultations.

The therapy management group could for example withdraw its support or assistance to the client for lack of information about his health condition to decide on how he can be helped. It may be for this reason that Mellvray and MacClean as cited in Janzen [10] exhort clinicians working in hospitals and medical centres in Africa to be sensitive to their patients’ therapy managers and to share information with them. Sharing information is likely to enlist the full support of the group towards the effort to find a cure or treatment for patients. The advice by Mellvray and MacClean also shows that in sub-Saharan Africa, it takes the combined effort of health workers and a client’s therapy management group to successfully cure or treat a patient of his sickness. The assumption is that when information about the patient’s health problem or sickness is shared with the group, rather than maintaining confidentiality, the patient gets a decent treatment beyond a clinical decision. The present study suggests that maintaining confidentiality in the care and treatment of HIV may complicate the work of care providers and also create problems for clients due to the crucial role of the therapy management group.

Another issue emerging from the data presented in this paper has to do with the debate of either providing HIV-related care and treatment services in isolation of or integrated with other services in the hospitals. The debate has been necessitated by the obvious preference of clients for centres and clinics sited in discrete or obscure locations in the hospitals due to the persistent stigma associated with the disease. In essence, the debate for and against integration or isolation by health workers is basically aimed at conveniently providing services to clients so they could use services in privacy and conceal their status from relatives and friends. Both arguments have merits and demerits which call for critical examination by health workers. It is therefore the responsibility of health care professionals to decide whether integration should be ‘partial’ or ‘complete’. In the case of partial integration, health workers would have to make it clear which aspects of counselling, testing and treatment need to be provided alongside other services, and in which specific units. With regard to total integration, it is important for health workers to identify which particular unit or units such services could be conveniently provided for clients to feel secure and to continue to access services. If isolation is chosen, it would be necessary to find locations in the hospitals which would be appropriate for such services. Perhaps, the locations for such services could be tucked away from open places to prevent other patients from identifying clients while they use services. This observation is in line with an assertion made by Odeny [20] in a study carried out in rural Kenya on integration of HIV care with primary health care services. The authors pointed out that stigma may be reduced if a patient’s HIV status cannot be determined by the general public simply by observing the physical location where a patient is receiving care. In the event that health workers decide to integrate HIV-related health care with other services, then they may consider putting up new facilities at obscure locations in spite of the perennial lack of funds. However, Odeny [20] opined that integration of services may lead to other opportunities for accidental disclosures of one’s HIV status which can be a barrier to using services. For instance, a clients’ status could be known to another patient who is not HIV-positive in the process of accessing care in a facility which has an integrated service delivery. In this regard, the effect of integration on stigma is unclear.

The debate on integration or non-integration of HIV counselling testing and treatment with other health care services finds meaning in a study conducted in Kenya and Swaziland by Population Council [21] and three other non-governmental organizations on the integration of sexual and reproductive health and HIV services. Conducted from 2008 to 2013, the Integration Initiative sought to generate rigorous evidence on feasibility, effectiveness, cost, and impact of different models for delivering integrated HIV/AIDS services in settings with high and medium HIV prevalence in sub-Saharan Africa. The study demonstrated that most clients prefer fully integrated services to save time and money and it also reduce the fear of stigma because clients trusted the providers of such facilities. Similarly, Baotran [22] indicated that the findings of their study on patient satisfaction with integrated HIV and antenatal care services in rural Kenya suggests that integration is highly acceptable to most HIV-infected women. According to them, integration improved HIV-infected women’s satisfaction with overall clinic experience. They attributed women’s satisfaction with integration to the ease of receiving care for both services in one single visit, removing the logistical difficulties of standing in line for services at two different clinics, and the possible disclosure of their status to other non HIV-positive persons. It is for this reason that some health workers in this study also advocated for the integration of HIV-related services into other care services in order to address the concerns of clients who in most cases are more worried about people knowing their identity as PLWAs than care and treatment they would receive in the facilities.

Whichever option health workers or planners decide to go for, one important thing that they must not forget is the interest and welfare of clients who are the ultimate beneficiaries of health care services. Integration or isolation of the services must ensure that clients are guaranteed privacy in the use of services to avoid identification in the health care setting and prevent stigmatisation. This is likely to encourage clients to continue accessing services and, also motivate potential clients to use services.

## Conclusion

This article has shown that clients tend to be more concerned about the social risks of treatment than about the medical outcomes of accessing counselling, testing and treatment services. The concern is largely due to some perceived individual and institutional or structural-level gaps in the health care setting. Under normal circumstances, patients accessing health care services are interested in the required cure or treatment for their health problems, with little interest in whether someone they know may see them in the hospital. However, the same cannot be said of HIV care and treatment because of the disease’s peculiar status. It has therefore become necessary for its sufferers to use services in confidentiality and privacy to hide their status from relatives, spouses and others, and avoid stigmatisation. The disease is here to stay with the people and the health care delivery system calls for a comprehensive look at the way its services are provided and used. The concerns of clients must be taken into consideration in whichever way the services are provided or would be provided in the future, to ensure success when the system is made more widespread. The inability of health care authorities to effectively do this may be a missed opportunity for encouraging more clients and potential clients to access counselling, testing and treatment services in the effort to reduce the spread of the disease.

More importantly, from the encounters between the sero-positives and health personnel, it is undeniably clear that the former’s rights as enshrined in the Patients’ Charter have been grossly violated. This finding may not be an isolated case, for the rights of patients and the responsibility of health personnel have remained a moot point since the beginning of the twentieth century when allopathy began to make in-roads into the minutiae of people’s daily lives. In the case of Ghana and of the research community for that matter, there appears to be a conflict between the professional norms and values of the health personnel and the moral need to protect the health of the larger community. In this conflicted situation, the dominance of health personnel and their ability to flout their own laws have been accentuated by weak oversight structures, the community’s mass illiteracy and poverty, the poor social space in which the AIDS-infected negotiate their daily lives and by the nature of HIV/AIDS itself. Clearly, a massive uptake of anti-retroviral and HIV/AIDS-related services will depend to a large extent on the degree to which Ghanaian health personnel respect not only the Hippocratic Oath, but also the Patient’s Charter.

## Abbreviations

AIIDS, Action Program; ART, Antiretroviral Therapy; HRSA, Health Resources and Services Administration; IAE, Institute of Adult Education; MOH, Ministry of Health, Ghana; SIAAP, South India AIDS Action Group; UG, University of Ghana; UNF, United Nations Population Fund; VCT, Voluntary Counselling and Testing.

## Additional file

Additional file 1: Interview guide. Description; This was the data collection instrument which was used during in-depth interviews to collect information from health workers and patients who participated in the study. (DOCX 4.99 kb)
