# Supplementary material for: HIV/AIDS clients, privacy and confidentiality; the case of two health centres in the Ashanti Region of Ghana
Source: BMC Med Ethics. 2016 Jul 16;17:41. doi: 10.1186/s12910-016-0123-3 (PMC4947355; doi:10.1186/s12910-016-0123-3)
Supplement: Additional file 1: — Interview guide. Description; This was the data collection instrument which was used during in-depth interviews to collect information from health workers and patients who participated in the study. (DOCX 4.99 kb) [file 12910_2016_123_MOESM1_ESM.docx]

Part 1

INTERVIEW GUIDE FOR VOLUNTARY COUNSELLING (VCT)/ANTIRETROVIRAL THERAPY (ART) HEALTH WORKERS

1. To what extent do people/clients access services in the Voluntary Counselling and Testing (VCT)/Antiretroviral Therapy (ART) center?

2. Describe the processes clients go through when they access VCT/ART.

3, How would you describe the quality of services you provide to VCY/ART clients in this facility?

4. How do health workers keep information about clients confidential?

5. How is the privacy of clients ensured during the up-take of VCT/ART services?

6. How do health workers interact with and relate to VCT/ART clients?

7. What challenges do you face in the provision of services in this facility?

8. Suggest ways the challenges you face can be solved to ensure the provision of high quality care and treatment.


Part 2

INTERVIEW GUIDE FOR VOLUNTARY COUNSELLING AND TESTING (VCT)/ANTIRETROVIRAL THERAPY (ART) CLIENTS

1. Describe how you got HIV/AIDS infection.

2. What was your initial reaction when the counselor first disclosed that you have tested HIV-positive?

3, Generally, how have you managed to live with the HIV-positive status?

4. Describe how health workers interact with and relate to VCT/ART clients? Probe on attitudes and behaviours of health workers

5, How would you describe the quality of services you receive in this facility? Probe on confidentiality and privacy.

6. Why do you think some people refuse to voluntarily take up HIV/AIDS counseling and testing? Probe on confidentiality

7. What challenges do you face in accessing care and treatment in this facility? Probe on location of facilities.

8. Suggest ways in which the challenges you face can be solved.
